# ﻿Morpho-phylogenetic analyses of two novel edible mushrooms from China and a mini review of *Lyophyllum* (Agaricales, Lyophyllaceae) cultivation and bioactivities

**DOI:** 10.3897/mycokeys.112.141615

**Published:** 2025-01-23

**Authors:** Song-Ming Tang, Feng-Ming Yu, Samantha C. Karunarathna, Zong-Long Luo, Kai-Yang Niu, Rui-Yu Li, Lin Li, Xi-Jun Su, Shu-Hong Li

**Affiliations:** 1 College of Agriculture and Biological Science, Dali University, Dali 671003, China Dali University Dali China; 2 Key Laboratory for Plant Diversity and Biogeography of East Asia, Yunnan Key Laboratory of Fungal Diversity and Green Development, Kunming Institute of Botany, Chinese Academy of Sciences, Kunming 650201, Yunnan, China Kunming Institute of Botany, Chinese Academy of Sciences Kunming China; 3 Center for Yunnan Plateau Biological Resources Protection and Utilization, College of Biology and Food Engineering, Qujing Normal University, Qujing, Yunnan 655011, China Qujing Normal University Qujing China; 4 Biotechnology and Germplasm Resources Institute, Yunnan Academy of Agricultural Sciences, Kunming 650205, China Biotechnology and Germplasm Resources Institute, Yunnan Academy of Agricultural Sciences Kunming China

**Keywords:** 2 new species, Agaricales, edible mushroom, *Lyophyllumshimeji*, multi-gene phylogeny

## Abstract

*Lyophyllum* plays an important role in the natural ecosystem and has significant economic value. Some species of this genus have been cultivated in Asia, America, and Europe. This study describes four edible species of *Lyophyllum*, two of which were newly discovered. *Lyophyllumedulis* has a dark grayish orange pileus, a grayish orange upper part of the stipe, and globose, subglobose to broadly ellipsoid basidiospores, while *L.sinense* has a dark gray-orange when injured pileus, dark grayish orange points and lines on the stipe surface, and quadrangular to broadly fusiform basidiospores. Molecular phylogenetic analyses using the internal transcribed spacer ITS1-5.8S-ITS2 ribosomal RNA (ITS), the large subunit ribosomal RNA (LSU), the second-largest subunit of RNA polymerase II (*rpb2*), and translation elongation factor 1-alpha (*tef1-α*) indicated that *L.edulis* is related to *L.shimeji*, *L.heimogu*, and *L.decastes*, and *L.sinense* has an affinity to *L.bulborhizum* and *L.nigrum*. We also summarize the cultivation techniques of the two edible species, *L.shimeji* and *L.decastes*.

## ﻿Introduction

*Lyophyllum* P. Karst. was established based on the type species, *L.leucophaeatum* (P. Karst.) P. Karst. (Karsten 1881). *Lyophyllum* species are characterized by variable and complex, basidiomata clustered or scattered, basidiospores globose, oblong, or broadly fusiform (Clémençon and Winterhoff 1992; Vizzini and Contu 2010); the lamellae and stipe of some species change to dark gray-orange when injured (Wang et al. 2013); the culture texture is smooth, velvet, or cotton (Arana-Gabriel et al. 2018).

To date, approximately 70 species of *Lyophyllum* have been described worldwide (He et al. 2019; Zhang et al. 2021; Ma et al. 2022, 2023; Li et al. 2023, 2024; Wei et al. 2023), of which 24 species have been reported in China, among them *L.bulborhizum* S.M. Tang & S.H. Li, *L.decastes* (Fr.) Singer, *L.deqinense* S.H. Li, *L.heimogu* S.H. Li, and *L.pallidofumosum* Y.H. Ma, W.M. Chen & Y.C. Zhao, *L.shimeji* (Kawam.) Hongo, and *L.yiqunyang* S.H. Li. (Li et al. 2010; Feng et al. 2019; Zhang et al. 2021; Ma et al. 2022; 2023; Li et al. 2023, 2024; Wei et al. 2023) are widely edible.

*Lyophyllum* species have previously been placed in several genera such as *Agaricus* (Britzelmayr 1881), *Hygrophorus* (Boudier 1878), *Collybia* (Karsten 1889), and *Tephrocybe* (Orton 1988). Some species from different genera have mistakenly been placed in *Lyophyllum*, such as *L.albellum* (Fr.) Consiglio & Contu as *Calocybealbella* (synonym) (Bon 1995), *L.ambustum* (Fr.) Singer as *Tephrocybeambusta* (Fr.) Donk (synonym) (Donk 1962), and *L.albofloccosum* (P.D. Orton) Consiglio & Contu as *Myochromellaboudieri* (synonym) (Kühner & Romagn.) V. Hofst., Clémençon, Moncalvo, and Redhead (Hofstetter et al. 2014), making the taxonomy of *Lyophyllum* confus­ing and difficult to understand.

The species of *Lyophyllum* currently being commercially cultivated include *L.shimeji* and *L.decastes* (Akamatsu 1998; Yamada et al. 2001; Boa 2005; Pokhrel et al. 2006), despite *L.shimeji* having been described as a form of facultative mycorrhiza (Ohta 1994) and *L.decastes* also having been described as ectomycorrhizal with *Pinuspinaster* (Pera and Alvarez 1995). With the development of biomedicine, several species of *Lyophyllum* have been developed and utilized. *Lyophyllumdecastes* exhibits many biological activities, including antitumor, radioprotective (Iwasa et al. 2006), antidiabetic (Miura et al. 2002), antifungal, anticholinesterase, and antioxidant effects (Tel et al. 2015).

Recently, molecular phylogenetic approaches have been increasingly applied to investigate the phylogenetic relationships among the genera and Lyophyllaceae species (Li et al. 2023, 2024; Ma et al. 2022, 2023; Tang et al. 2023; Wei et al. 2023). These studies have effectively enriched the diversity of the Lyophyllaceae. Over the past decade, the application of molecular biology has significantly expanded our knowledge of the Lyophyllaceae, particularly the species of *Termitomyces* and *Lyophyllum* (Li et al. 2023, 2024; Ma et al. 2022, 2023; Tang et al. 2023; Wei et al. 2023). However, the majority of phylogenetic analyses are based solely on ITS or ITS and LSU, leaving the relationships among species unclear, since there are fewer variable sites in ITS and LSU between different species. This underscores the need for further research to fully understand the phylogenetic relationships within the Lyophyllaceae.

In this study, we conducted a comprehensive investigation of *Lyophyllum* across China, resulting in the discovery and description of two novel and two known species of *Lyophyllum*. Our findings, supported by molecular phylogenetic analyses based on ITS1-5.8S-ITS2, LSU, *rpb*2, and *tef*1-α genes, significantly contribute to the classification and understanding of *Lyophyllum* species.

## ﻿Materials and methods

### ﻿Morphological studies

Macromorphological characteristics and habitat descriptions were obtained from photographs and field notes. Color identification was performed using the Color Hexa website (www.colorhexa.com) to assign codes. After recording the macromorphological characteristics, the specimens were dried at 45–50 °C (Hu et al. 2022) in a food dehydrator until no more moisture was left. The dried specimens were then stored in sealed plastic bags. In the microscopic study, we conducted a thorough examination of the dried mushroom materials. They were sliced and placed in a 5% KOH solution and 1% Congo red for mounting. Microscopic features such as basidia, basidiospores, and cystidia were meticulously examined and photographed using a light microscope (Nikon Eclipse 80i, Japan). In the descriptions of microscopic characters, measurements were conducted on 50–100 basidiospores and 20 basidia and cystidia randomly selected; acetoferric carmine was also used to check the siderophilous granulations in the basidia (Kühner 1938). The notation [x/y/z] indicates × basidiospores measured from y basidiomata of the z collection. Basidiospore dimensions are denoted as (a–) b–c (–d), where the range b–c represents 95% of the measured values, and “a” and “d” are extreme values. Q refers to individual basidiospore length/width ratio, while Q_m_ refers to the average Q value ± standard deviation. The specimens were stored in sealed plastic bags and deposited in the Herbarium of Cryptogams, Kunming Institute of Botany, Academia Sinica (KUN-HKAS).

### ﻿DNA extraction, PCR amplification, and sequencing

Genomic DNA extraction from dry specimens was performed using the Ezup Column Fungi Genomic DNA Extraction Kit (Genesand Biotech Co., Ltd., Beijing, China) according to the manufacturer’s protocol. Subsequent steps included PCR amplification, PCR product purification, and sequencing. The primer pairs used for PCR were ITS1/ITS4 (White et al. 1990), LR5/LR0R (Vilgalys and Hester 1990), *rpb*2-5F/*rpb*2-7cR (Liu et al. 1999), and *tef*1-α 983F/*tef*1-α 2218R (Rehner and Buckley 2005). PCR was executed on a C1000 Thermal Cycler (Bio-Rad) with the following cycling program for ITS and LSU: initial denaturation at 94 °C for 5 min, 35 cycles of denaturation at 94 °C for 30 s, annealing at 48 °C for 30 s, extension at 72 °C for 90 s, and a final extension at 72 °C for 10 min; for *tef*1 and *rpb*2: initial denaturation at 95 °C for 5 min, 35 cycles of denaturation at 95 °C for 30 s, annealing at 55 °C for 30 s, extension at 72 °C for 90 s, and a final extension at 72 °C for 10 min.

### ﻿Sequence alignment and phylogenetic analyses

The sequences of *Lyophyllum* species obtained in this study (Table 1), along with sequences from phylogenetic analyses (Hofstetter et al. 2002; Bellanger et al. 2015; Li et al. 2023), were aligned using MAFFT version 7 (Katoh and Standley 2013) and verified using BioEdit version 7.0.5 (Hall 2011). Consistent with previous phylogenetic investigations, *Calocybeionides*, *C.carnea*, and *C.persicolor* were employed as outgroup taxa (Hofstetter et al. 2002).

**Table 1.** Names, voucher numbers, origins, and corresponding GenBank accession numbers of taxa used in the phylogenetic analyses. Newly generated sequences are shown in bold. “*” following a species name indicates that the specimen is the type of that species, and “N/A” refers to the unavailability of data.

Taxon name	Voucher numbers	Origin	GenBank accession no.			
			ITS	LSU	*rpb*2	*tef*1-α
*Lyophyllumbulborhizum*	L5083*	China	PP406873	**PQ471271**	**PQ523769**	**PQ533687**
*L.bulborhizum*	L5092	China	PP406874	**PQ471272**	**PQ523770**	**PQ533688**
*L.edulis*	HKAS 135644*	China	**PQ471283**	**PQ471265**	**PQ523777**	**PQ533681**
*L.edulis*	HKAS 135645	China	**PQ471284**	**PQ471266**	**PQ523776**	**PQ533682**
*L.heimogu*	L3033	China	KY434101	**PQ471276**	**PQ523783**	**PQ533690**
*L.heimogu*	L3035	China	KY434102	**PQ471277**	**PQ523784**	**PQ533691**
*L.heimogu*	L3026*	China	KY434100	**PQ471278**	**PQ523782**	**PQ533689**
*L.nigrum*	L5186	China	PP406877	**PQ471274**	**PQ523774**	**PQ533693**
*L.nigrum*	L5091*	China	PP406876	**PQ471273**	**PQ523773**	**PQ533692**
*L.nigrum*	L5187	China	PP406878	**PQ471275**	**PQ523775**	**PQ533694**
*L.pallidofumosum*	HKAS 135649	China	**PQ471287**	**PQ471269**	**PQ523780**	**PQ533685**
*L.pallidofumosum*	HKAS 135650	China	**PQ471288**	**PQ471270**	**PQ523781**	**PQ533686**
*L.pallidofumosum*	L5099	China	**PQ471279**	**PQ471261**	**PQ523767**	**PQ533677**
*L.pallidofumosum*	L5100	China	**PQ471280**	**PQ471262**	**PQ523768**	**PQ533678**
*L.rhombisporum*	L5010	China	PP406879	N/A	**PQ523772**	**PQ533695**
*L.rhombisporum*	L5084	China	PP406880	N/A	**PQ523771**	**PQ533696**
*L.sinense*	HKAS 144417*	China	**PQ471281**	**PQ471263**	**N/A**	**PQ533679**
*L.sinense*	HKAS 144418	China	**PQ471282**	**PQ471264**	**N/A**	**PQ533680**
*L.shimeji*	HKAS 135647	China	**PQ471285**	**PQ471267**	**PQ523778**	**PQ533683**
*L.shimeji*	HKAS 135648	China	**PQ471286**	**PQ471268**	**PQ523779**	**PQ533684**

Phylogenies and node support were initially deduced through maximum likelihood (ML) using RAxML-HPC2 version 8.2.12 (Stamatakis 2014). This process involved separate analyses of three single-gene alignments with 1,000 rapid bootstraps and was executed on the Cipres portal (Miller et al. 2010). Since there was no identified conflict with substantial support [bootstrap support value (BS) ≥ 70%] among the topologies, the four single-gene alignments were concatenated using Sequence Matrix (Vaidya et al. 2011). For partitioned maximum likelihood (ML), the concatenated dataset was analyzed following the previously mentioned procedure (Stamatakis 2014). For Bayesian Inference (BI), the optimal substitution model for each character set was identified using MrModeltest version 2.3 (Nylander et al. 2004) on the CIPRES (https://www.phylo.org/) platform. The four partitions selected models were GTR+I for ITS1+ITS2, TrN + I + G for LSU + 5.8S, JC + I + G for the *rpb*2 exon + *tef*1-α exon, and F81 + G for the *rpb*2 intron + *tef*1-α intron. Bayesian analysis was performed using MrBayes version 3.2.7a (Ronquist et al. 2011) as implemented on the Cipres portal (Miller et al. 2010), in which two runs of six chains each were conducted by setting generations to 500,000 and the stoprul command with the stopval set to 0.01, and trees were sampled every 200^th^ generation. A clade was considered strongly supported if BS ≥ 70% and posterior probability (PP) ≥ 0.90. The alignment was submitted to Figshare (10.6084/m9.figshare.27117543).

## ﻿Results

### ﻿Phylogenetic analyses

In the phylogenetic analysis, 68 new sequences were included, generated from 20 specimens, with other sequences referring to the study (Li et al. 2023; Hofstetter et al. 2002; Bellanger et al. 2015). After trimming, the ITS1 + ITS2, LSU + 5.8S, *rpb*2 exon + *tef*1-α exon, and *rpb*2 intron + *tef*1-α intron sequences had 252, 1,235, 1,546, and 170 characters, respectively. The combined dataset had an aligned length of 3,203 characters, of which 721 were constant, 1,142 were variable but parsimony-uninformative, and 931 were parsimony-informative.

**Figure 1.**
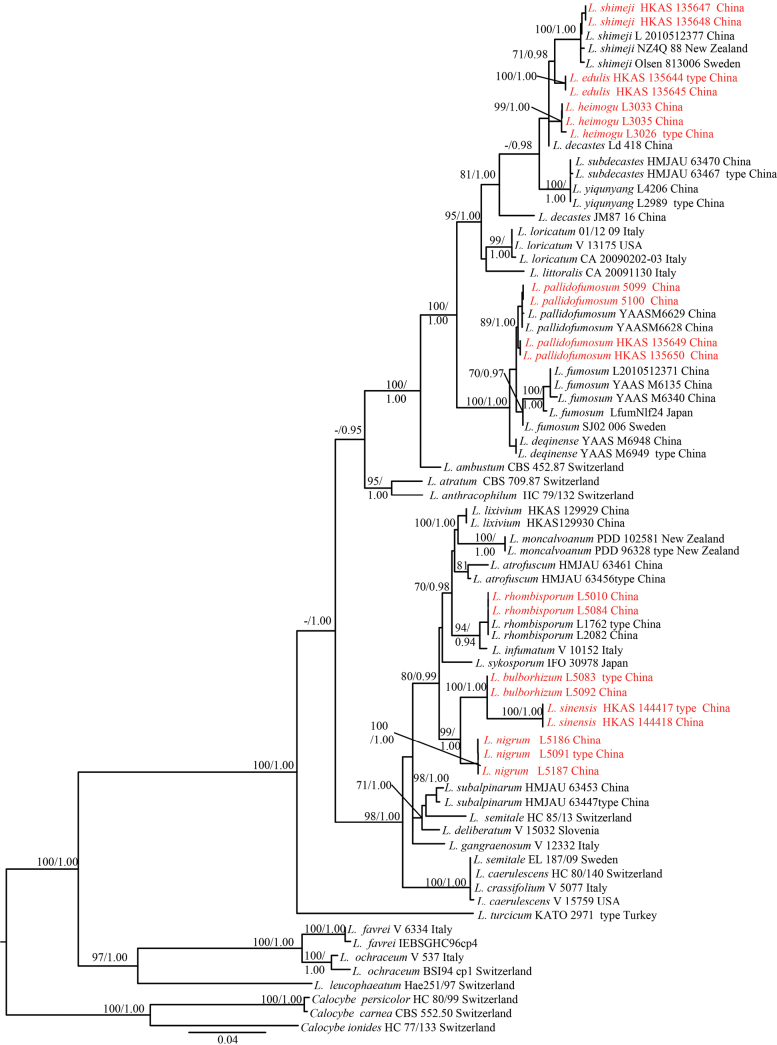
Strict consensus tree illustrating the phylogeny based on the combined ITS1 + ITS2, LSU + 5.8S, *tef*1 exon + *rpb*2 exon, and *tef*1 exon + *rpb*2 intron datasets. Maximum likelihood bootstrap proportions equal to or higher than 70% and Bayesian posterior probabilities equal to or higher than 0.90 are indicated at nodes. *Calocybeionides*, *C.carnea*, and *C.persicolor* were used as outgroup taxa. The sequences generated in this study are in red.

ML and BI analyses generated nearly identical tree topologies, with little variation in statistical support. Therefore, only the ML tree is shown (Fig. 1). Phylogenetic data, together with thorough morphological analysis (see below), showed that the two newly described taxa in this study were significantly different from other known *Lyophyllum* species.

### ﻿Taxonomy

#### ﻿ Lyophyllum edulis

Taxon classificationFungiAgaricalesLyophyllaceae

S.M. Tang & S.H. Li sp. nov.

983CC7D6-9DF8-5C61-993A-CBA3AEFC15CA

855910

Figs 2A, B , 3 , 4

##### Etymology.

The epithet “edulis” refers to the edibility of this species; locally it is considered a delicacy.

##### Holotype.

China, Sichuan Province: Jiuzhaigou County, elev. 2,100 m, October 12, 2023, Song-Ming Tang, L6737 (HKAS 135644!).

##### Description.

***Pileus*** 3–8 cm diameter, fleshy, fragile, hemispherical, becoming convex with age, smooth on the surface, dry, dark grayish orange (#8a7971) on the center, soft orange (#e9c7a7) with margin, subumbonate of center, inflexed of margin; pileus context thick, 0.2–0.3 cm wide, white (#fcfcfc). ***Lamellae*** moderately close together, arcuate, subdecurrent to decurrent, broad, white (#fcfcfc), unchanging color when injured, 3–4 tiers, 0.4–0.5 cm wide, edge even or entire. ***Stipe*** 3.7–6.9 × 0.8–1.4 cm, cylindrical, grayish orange (#d9cdc2) in the upper, soft orange (#e9c7a7) gradually downward, smooth; stipe context white (#fcfcfc), solid, unchanging in color when injured. The odor and taste were not distinctive.

**Figure 2.**
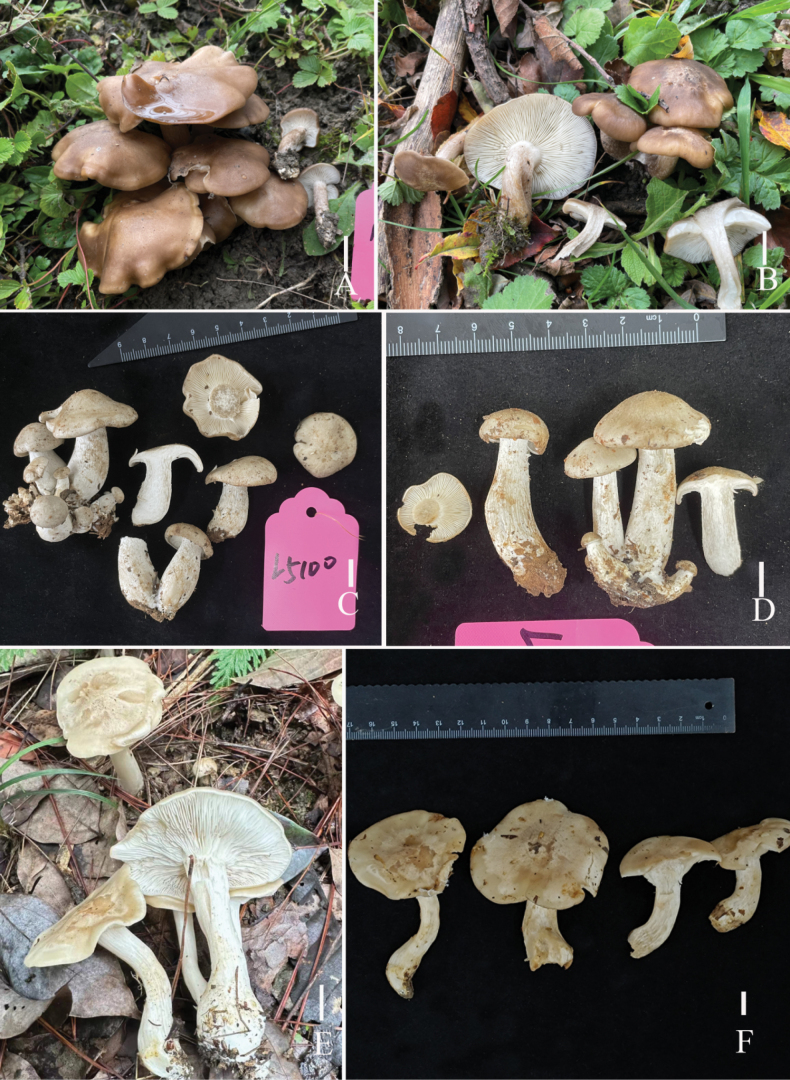
Fresh basidiomata of the two new *Lyophyllum* species **A, B***L.edulis* (A L6737 holotype, B L6741) **C–F***L.pallidofumosum* (**C** L5100, D L5099, **E, F** L6883). Scale bars: 1 cm.

***Basidiospores*** [84/2/2] 5.1–6.5 (–8) × 4.6–6.6 μm, (Q = 1.0–1.2, Qm = 1.11 ± 0.05), av. 5.81 ± 0.28 × 5.47 ± 0.38 μm, globose, subglobose to broadly ellipsoid, hyaline, smooth. ***Basidia*** 25–39 × 8–11 μm (N = 20), av. 32.7 ± 5.1 × 9.7 ± 1.0 μm, mostly 4-spored, rarely 2-spored, sterigmata long 1.8–4.9 μm, sometimes with basal clamp connections, clavate, siderophilous granulations. ***Subhymenium*** is composed of moderately thin-walled hyphae, 40–55 μm thick, with 2–3 layers of ovoid, fusiform to narrowly cylindrical hyphae, and 6–8 × 3–5 μm. ***Hymenophoral trama*** regular, 120–150 μm wide, consisting of thin and hyaline hyphae, some with clamp connections, narrowly cylindrical hyphal elements, 6–12 μm wide. ***Cheilocystidia*** were 21–24 × 4–7 μm in size and av. 22.9 ± 1.3 × 6.4 ± 0.7 μm, narrowly cylindrical or narrowly clavate, thin-walled, and rarely mucronate or rostrate on the apex. ***Pleurocystidia*** 24–28 × 4–6 μm, av. 26.3 ± 1.6 × 5.3 ± 0.6 μm, narrowly cylindrical or narrowly clavate, thin-walled. ***Pileipellis*** colorless and hyaline in 5% KOH solution, parallel, thin-walled, almost cylindrical to subcylindrical, filamentous hyphae 4–6 μm wide. ***Stipitipellis*** composed of appressed, parallel, thin-walled, hyphae 2–7 µm wide. ***Clamp connections*** are present at some septa in the pileipellis, lamellae, and stipitipellis.

**Figure 3.**
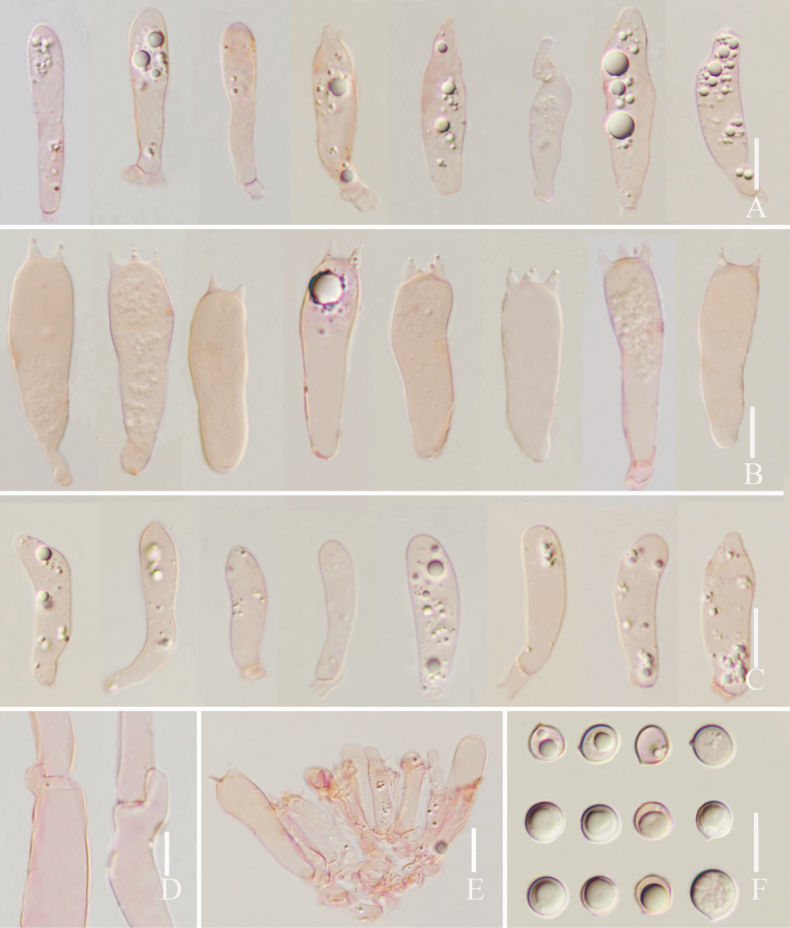
*Lyophyllumedulis* (L6737, HKAS 135644) **A** cheilocystidia **B** basidia **C** pleurocystidia **D** clamp connections **E** basidia and pleurocystidia **F** basidiospores. Scale bars: 10 µm.

##### Habitat.

Clustered, related to *Quercusglauca* in broad-leaved forests in Sichuan and Shandong provinces.

##### Economic value.

Edible, available in local markets.

##### Additional materials examined.

China • Sichuan Province: Jiuzhaigou County, elev. 2,380 m, October 12, 2023, Song-Ming Tang, paratype, L6738, HKAS 135645; Shandong Province, Jinan County, elev. 2,210 m, October 11, 2023, Tong Lv, L6880, HKAS 135646.

**Figure 4.**
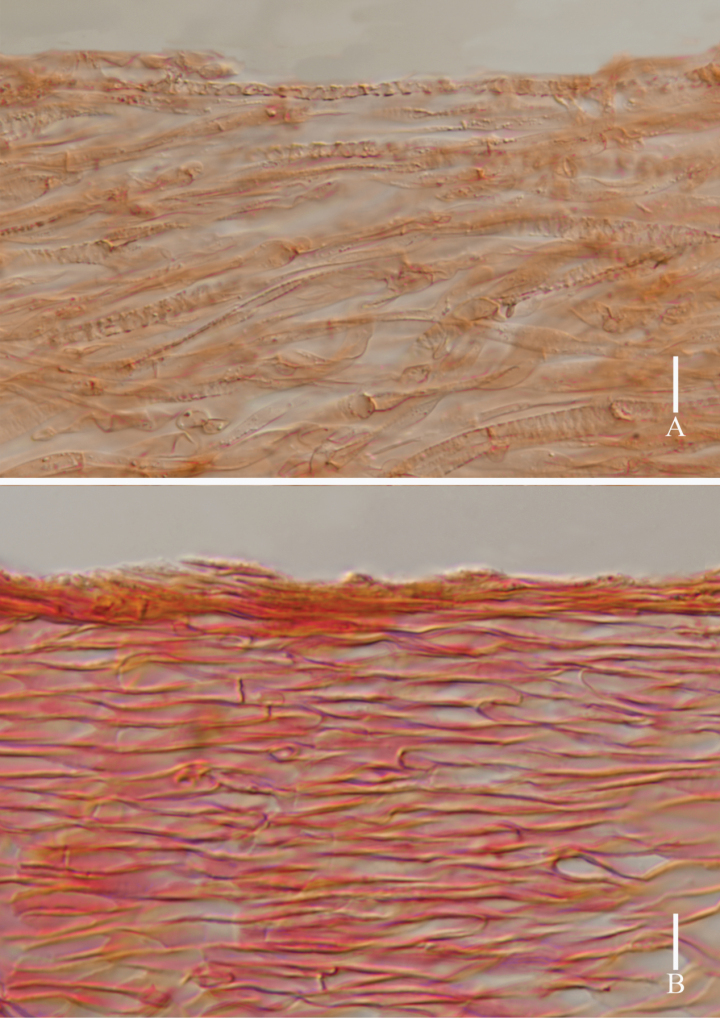
*Lyophyllumedulis* (holotype L6737) **A** pileipellis **B** stipitipellis. Scale bars: 10 μm.

##### Notes.

*Lyophyllumedulis* is similar to *L.fumosum*, *L.subdecastes*, *L.loricatum*, and *L.littorale* by sharing globose to subglobose basidiospores. However, the stipe surface of *L.fumosum* is cream to brown and has relatively larger basidia (40–45 × 8–10 µm; Sesli et al. 2015). *Lyophyllumsubdecastes* pileus surface is yellowish-brown or brown to greyish-red, stipe surface is reddish grey to greyish red, and smaller basidiospores (3.9–5.0 × 3.7–5.0 µm; Wei et al. 2023). *Lyophyllumloricatum* was originally described in Sweden; its pileus surface is reddish-brown to chestnut-brown, and the stipe surface is pale brownish or grey-brown (Breitenbach 1991). *Lyophyllumlittorale* stipe surface is grey and has smaller basidiospores (4.5–5.5 × 4.5–5.5 µm; Ballero and Contu 1990).

In our multi-locus phylogeny, *L.decastes* (Fr.) Singer, *L.shimeji* (Kawam.) Hongo, and *L.heimogu* S. H. Li are sister to the clade of *L.edulis.* However, the original description of *L.decastes* from Sweden has a whitish-greyish stipe (Breitenbach 1991; Trudell and Ammirati 2009; Davis et al. 2012), and ITS sequence differences between *L.edulis* (HKAS 135664, holotype) and *L.decastes* (Ld418) were 1.81% (10/552, including 2 gaps). *Lyophyllumshimeji*, originally described from Japan as *Tricholomashimeji* Kawam., has a dark grey to grey pileus; ITS sequence differences between *L.edulis* (HKAS 135664, holotype) and *L.shimeji* (L2010512377) were 4.89% (27/552, including 2 gaps). *Lyophyllumheimogu*, collected from Xizang, China, has dark grey to olive pileus and stipe surface yellowish-brown; ITS sequence differences between *L.edulis* (HKAS 135664, holotype) and *L.heimogu* (L3026, holotype) were 1.81% (10/552, including 2 gaps). Thus, they were classified as a heterospecific species.

#### ﻿ Lyophyllum pallidofumosum

Taxon classificationFungiAgaricalesLyophyllaceae

Y.H. Ma, W.M. Chen & Y.C. Zhao, in Ma, Liu, Zhao, Chen & Zhao, Phytotaxa 576(2): 178 (2022)

531101CA-8738-51DA-B0DF-17D6DDC079FC

Figs 2C–F , 5 , 6

##### Description.

***Pileus*** 2.0–6.0 cm diameter, fleshy, fragile, hemispherical, becoming convex with age, smooth on the surface, dry, grayish orange (#e4dfdb) on the center, soft orange (#dbcca9) with margin, slightly depressed to papilla of center, deflexed to inflexed of margin; pileus context thick, 0.2–0.3 cm wide, white (#fcfcfc). Lamellae moderately close together, arcuate, subdecurrent to decurrent, broad, white (#fcfcfc), unchanging color when injured, 2–3 tiers, 0.2–0.3 cm wide, edge even or entire. ***Stipe*** 4–7 × 0.9–1.1 cm, wide bulbous at the base, smooth; stipe context white (#fcfcfc), 1.2–3.0 cm wide, bulbous at the base, smooth; stipe context white (#fcfcfc), unchanging in color when injured. The odor and taste were not distinctive.

***Basidiospores*** [73/2/2] 4.5–6.6 × 4.0–5.9 μm, (Q = 1.0–1.3, Qm = 1.11 ± 0.10), av. 5.38 ± 0.59 × 4.89 ± 0.61 μm, globose, subglobose to broadly ellipsoid, hyaline, smooth. Basidia 19–28 (–35) × 10–15 μm (N = 20), av. 25.6 ± 4.1 × 12.1 ± 1.64 μm, mostly 2-spored, rarely 4-spored, sterigmata long 2.9–4.1 μm, sometimes with basal clamp connections, clavate, siderophilous granulations. ***Subhymenium*** is composed of moderately thin-walled hyphae, 15–20 μm thick, with 1–2 layers of ovoid, fusiform to narrowly cylindrical hyphae, 3–7 × 2–4 μm. ***Hymenophoral trama*** regular, 110–160 μm wide, consisting of thin and hyaline hyphae, some with clamp connections, narrowly cylindrical hyphal elements 2–5 μm wide. ***Cheilocystidia*** were 10–15 × 4–6 μm, av. 12.2 ± 1.8 × 5.0 ± 0.4 μm, narrowly cylindrical or narrowly clavate, rarely apex flexed, mostly narrowing with apex, thin-walled. ***Pleurocystidia*** were 12–18 × 4–6 μm in size and av. 14.8 ± 4.1 × 4.4 ± 1.1 μm, narrowly cylindrical or narrowly clavate, rarely apex flexed, mostly narrowing with apex, thin-walled. ***Pileipellis*** is an interwoven trichodermium composed of almost hyaline interwoven filamentous hyphae, terminal cells 2–5 μm wide, almost cylindrical to subcylindrical, occasional hyphal tips flexuous and sometimes inflate, and some with clamp connections. ***Stipitipellis*** composed of appressed, parallel, thin-walled, 2–4 µm wide, fusiform, thin-walled. ***Clamp connections*** are present at some septa in the pileipellis, lamellae, and stipitipellis.

**Figure 5.**
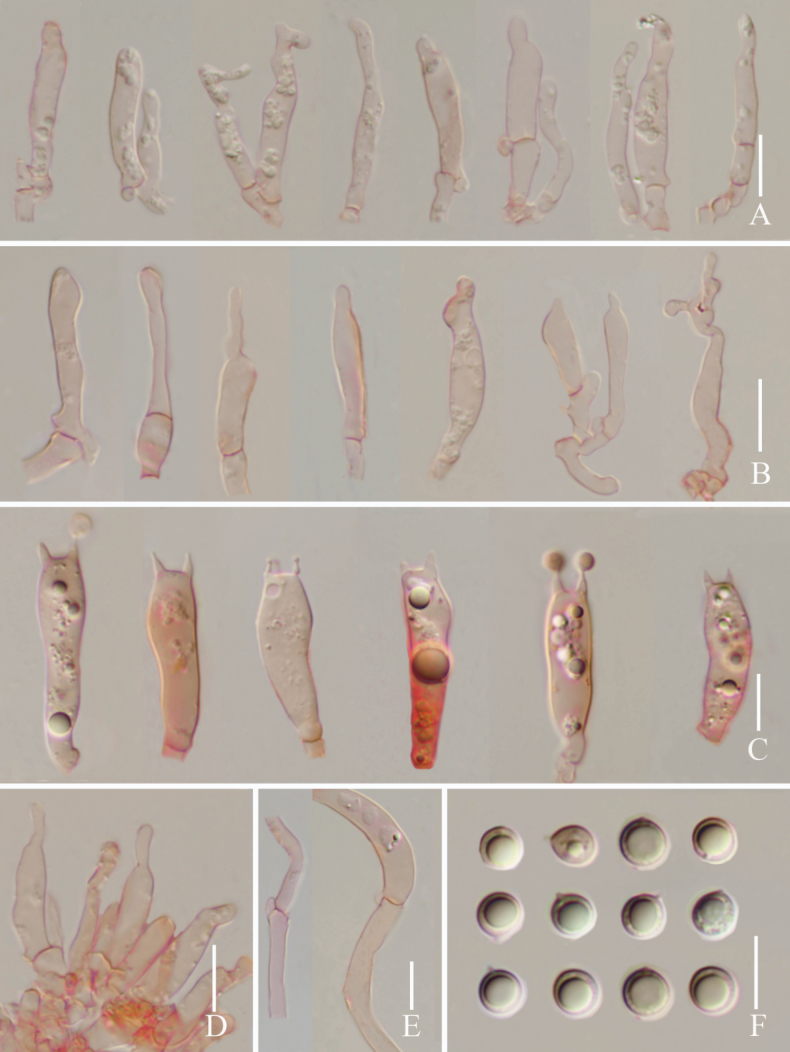
*Lyophyllumpallidofumosum* L6883 (HKAS 135649) **A** cheilocystidia **B** pleurocystidia **C** basidia **D** cheilocystidia **E** clamp connections **F** basidiospores. Scale bars: 10 μm.

##### Habitat.

Clustered, it occurs in the Sichuan and Yunnan provinces.

##### Additional species examined.

China • Chongqing Municipality, elev. 1,980 m, October 12, 2023, Song-Ming Tang, L6883, HKAS 135649; • Chongqing Municipality, elev. 2,152 m, L6884, October 12, 2023, Song-Ming Tang, HKAS 135650.

##### Notes.

*Lyophyllumpallidofumosum*, a new edible mushroom, has been published by Ma et al. (2022). However, the original description of *L.pallidofumosum* lacks cheilocystidia, pleurocystidia, pileipellis, and stipitipellis. Thus, in this study, we provide a more comprehensive description of *L.pallidofumosum*.

**Figure 6.**
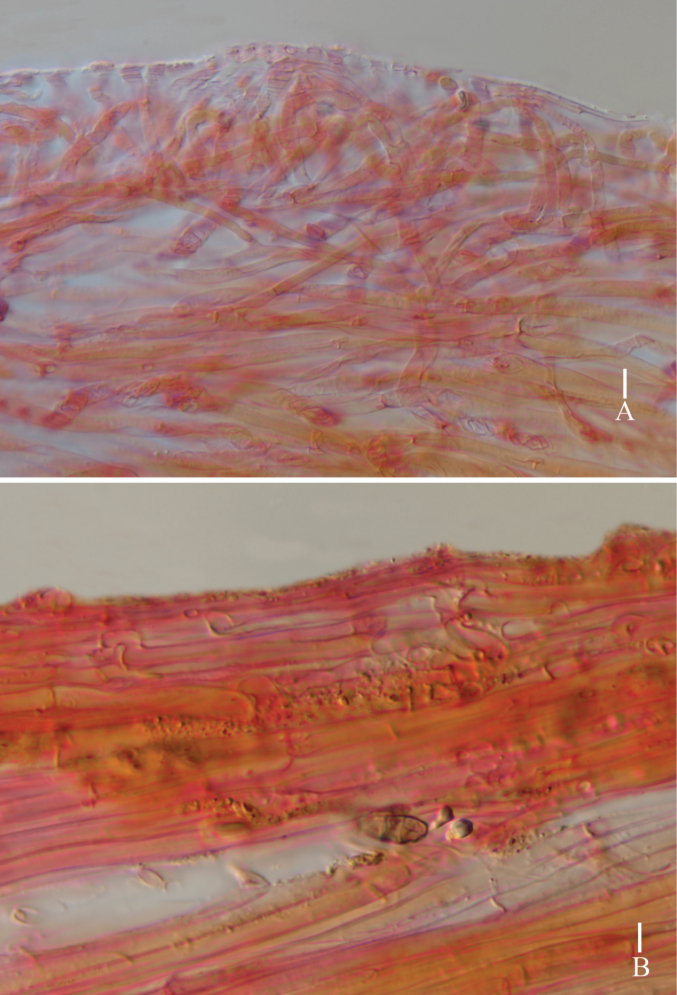
*Lyophyllumpallidofumosum* (L6883, HKAS 135649) **A** pileipellis **B** stipitipellis. Scale bars: 10 μm.

#### ﻿ Lyophyllum sinense

Taxon classificationFungiAgaricalesLyophyllaceae

S.M. Tang & S.H. Li sp. nov.

0B170E82-6D40-5461-909A-7F97218AE929

855911

Figs 7A, B , 8 , 9

##### Etymology.

The epithet “sinense” refers to the country “China,” where this fungus was first discovered.

##### Holotype.

China • Yunnan Province: Chuxiong Prefecture, Wuding County, elev. 2,119 m, 6 September 2023, Song-Ming Tang, L5090 (HKAS 144417!).

##### Description.

***Pileus*** 2.0–3.0 cm diameter, fleshy, fragile, hemispherical, becoming convex with age, abundant black floccus on the surface, dry, dark grayish orange (#a4a3a0) on the center, grayish yellow (#cac4b0) with margin, slightly depressed of center, involute of margin; pileus context thick, 0.3–0.5 cm wide, white (#fcfcfc). ***Lamellae*** moderately close together, arcuate, subdecurrent to decurrent, broad, white (#fcfcfc), grey dark orange (#a4a3a0) when injured, 3–4 tiers, 0.3–0.4 cm wide, edge even or entire. ***Stipe*** 3.0–4.0 × 0.9–1.8 cm, cylindrical to clavate, dark grayish orange (#a4a3a0) points and lines on the surface, bulbous at the base, smooth; stipe context white (#fcfcfc), changing to grayish orange (#c2bbab) when injured. The odor and taste were not distinctive.

**Figure 7.**
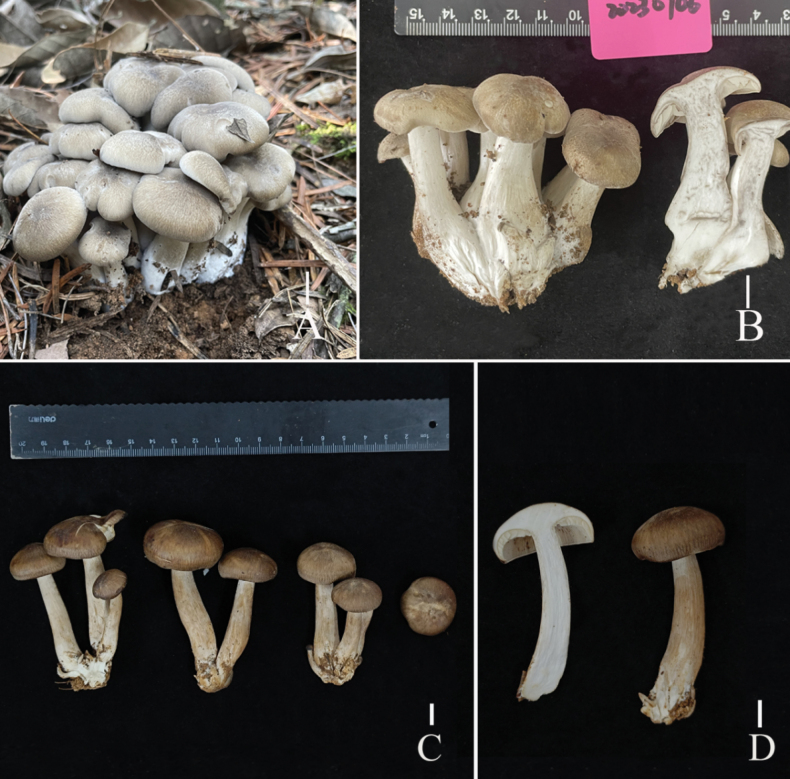
Fresh basidiomata of two *Lyophyllum* species **A, B***L.sinense* (L5090 holotype) **C, D***L.shimeji* (HKAS135647). Scale bars: 1 cm.

***Basidiospores*** [68/2/2] 6.1–8.6 × 5.5–7.1 μm, (Q = 1.0–1.3, Qm = 1.21 ± 0.12), av. 7.28 ± 0.68 × 6.07 ± 0.62 μm, quadrangular to very broadly fusiform, hyaline, smooth. Basidia 28–41 × 8–10 μm (N = 20), av. 34.6 ± 4.0 × 9.5 ± 0.53 μm, mostly 4-spored, rarely 2-spored, sterigmata long 2.2–3.9 μm, sometimes with basal clamp connections, clavate, siderophilous granulations. ***Subhymenium*** is composed of moderately thin-walled hyphae, 40–60 μm thick, with 2–3 layers of ovoid, fusiform to narrowly cylindrical hyphae, 5–7 × 2–4 μm. ***Hymenophoral trama*** regular, 130–180 μm wide, consisting of thin and hyaline hyphae, some with clamp connections, narrowly cylindrical hyphal elements, 4–7 μm wide. ***Cheilocystidia*** were 14–23 × 3–5 μm, av. 17.6 ± 2.4 × 4.1 ± 0.7 μm, narrowly cylindrical or narrowly clavate, thin-walled. ***Pleurocystidia*** were 10–25 × 3–6 μm in size and av. 17.2 ± 3.2 × 4.3 ± 1.1 μm, narrowly cylindrical or narrowly clavate, thin-walled. ***Pileipellis*** colorless and hyaline in 5% KOH solution, parallel, thin-walled, almost cylindrical to subcylindrical, filamentous hyphae 2–3 μm wide. ***Stipitipellis*** composed of appressed, parallel, thin-walled, hyphae 2–4 µm wide. ***Clamp connections*** are present at some septa in the pileipellis, lamellae, and stipitipellis.

**Figure 8.**
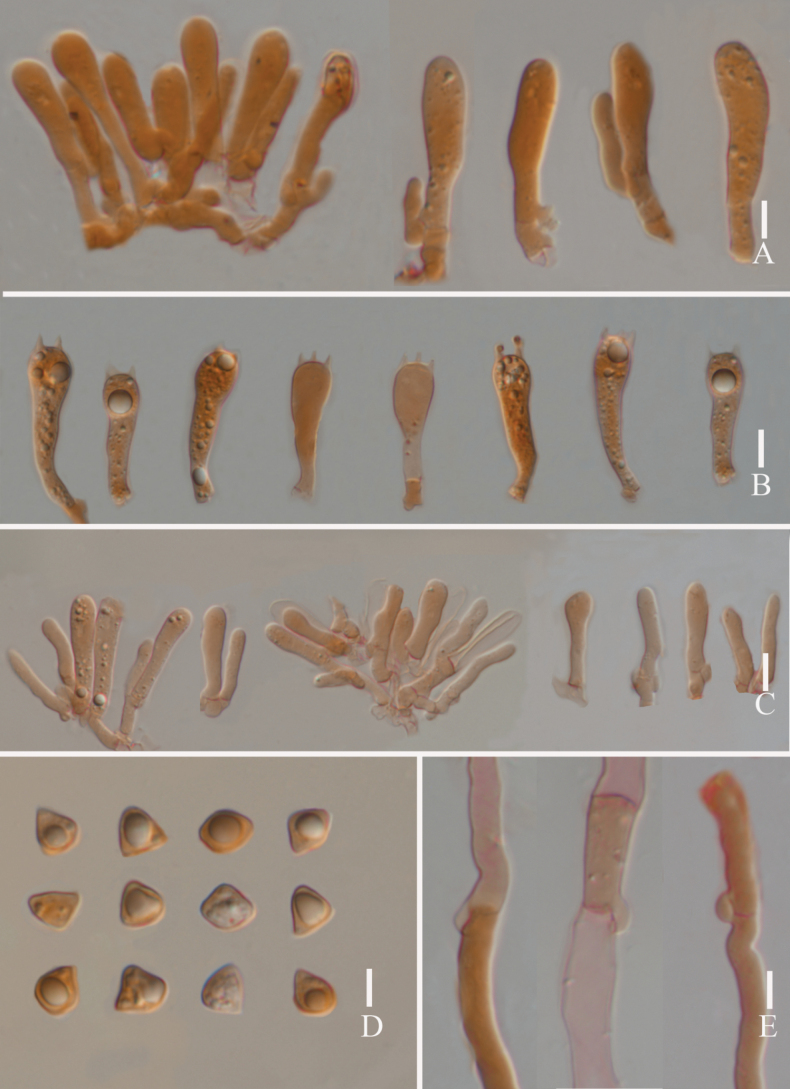
*Lyophyllumsinense* L5090 (HKAS 144417) **A** cheilocystidia **B** basidia **C** pleurocystidia **D** basidiospores **E** clamp connections. Scale bars: 10 μm.

##### Habitat.

Clustered in native forests in Yunnan, associated with *Lithocarpus* sp., at the base of the trees.

##### Edibility.

This species is an edible mushroom found in the Yunnan Province.

##### Additional species examined.

China • Yunnan Province, Chuxiong Prefecture, Wuding County, elev. 2,120 m, September 18, 2023, Song-Ming Tang, paratype, L5016, HKAS 144418.

##### Notes.

Morphologically, *L.sinense* is similar to *L.rhombisporum* and *L.subalpinarum*, with quadrangular to very broad fusiforms. However, *L.rhombisporum* has relatively longer cheilocystidia (28–40 × 5–8 µm) and pleurocystidia (20–46 × 4–6 µm) (Li et al. 2023). *Lyophyllumsubalpinarum*, which lacks cheilocystidia and pleurocystidia, has a grayish-yellow pileus and hollow stipe (Wei et al. 2023).

**Figure 9.**
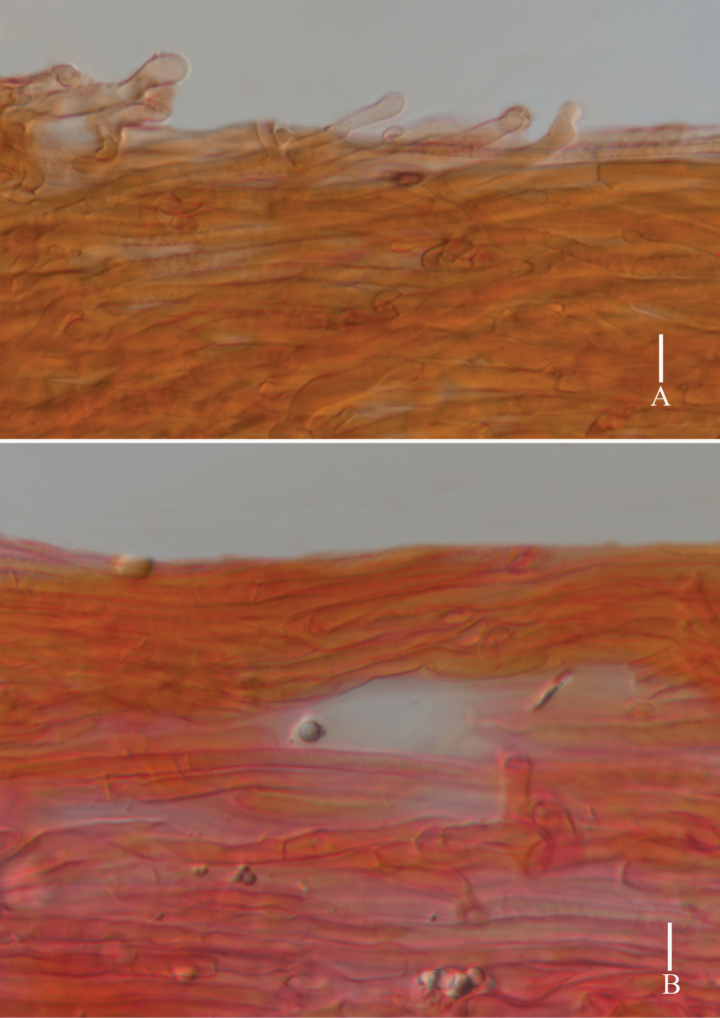
*Lyophyllumsinense* (L5090, HKAS 144417) **A** pileipellis **B** stipitipellis. Scale bars: 10 μm.

In our multi-locus phylogeny, *L.sinense* was found to be closely related to *L.bulborhizum* and *L.nigrum*. However, *L.bulborhizum*, mostly solitary, has a relatively bulbous at the stipe base; stipitipellis has abundant caulocystidia on the surface (Li et al. 2023). The ITS sequence difference between *L.sinense* (L5090, holotype) and *L.bulborhizum* (L5083, holotype) was 1.99% (11/552, not including gaps). *Lyophyllumnigrum* has relatively narrower lamellae (0.1–0.2 cm) and abundant caulocystidia on its surface (Li et al. 2023); the ITS sequence difference between *L.nigrum* (L5091, holotype) and *L.sinense* (L5090, holotype) was 3.62% (20/552, not including gaps).

#### ﻿ Lyophyllum shimeji

Taxon classificationFungiAgaricalesLyophyllaceae

(Kawam.) Hongo, Trans. Mycol. Soc. Japan 12(2): 90 (1971)

18303D58-D974-5E26-A17D-54C6D4C84675

**Figure 10.**
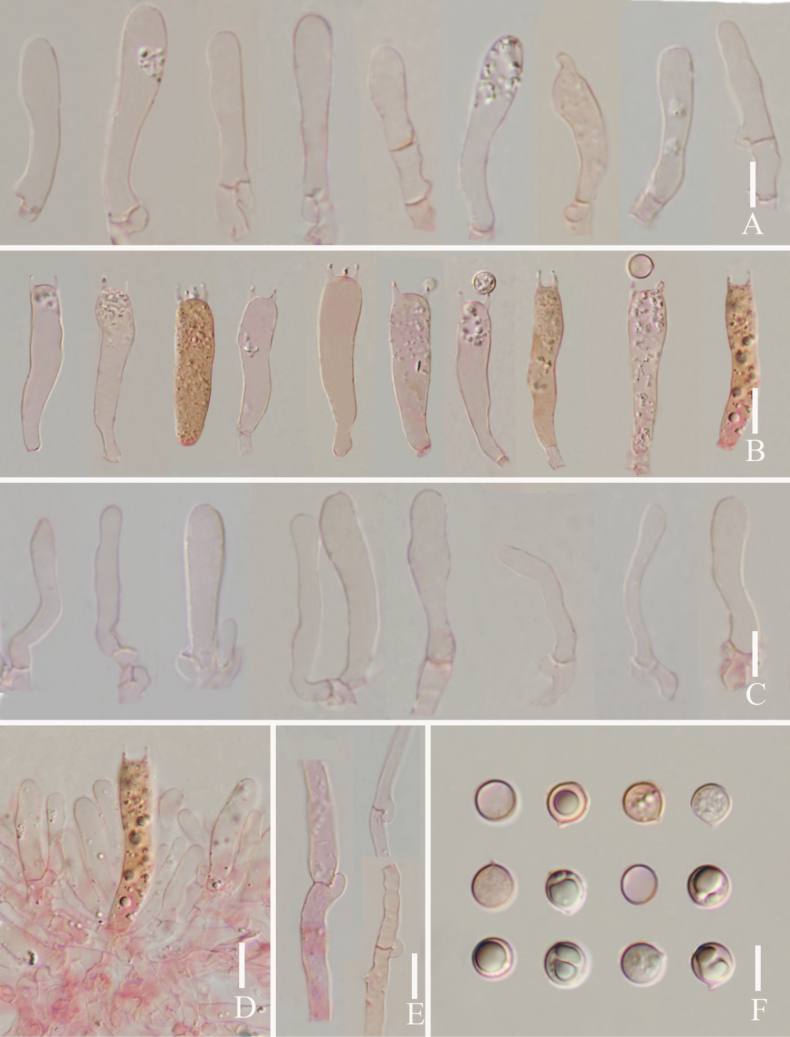
*Lyophyllumshimeji* (L6881) **A** cheilocystidia **B** basidia **C** pleurocystidia **D** basidia and pleurocystidia **E** clamp connections **F** basidiospores. Scale bars: 10 μm.

**Figure 11.**
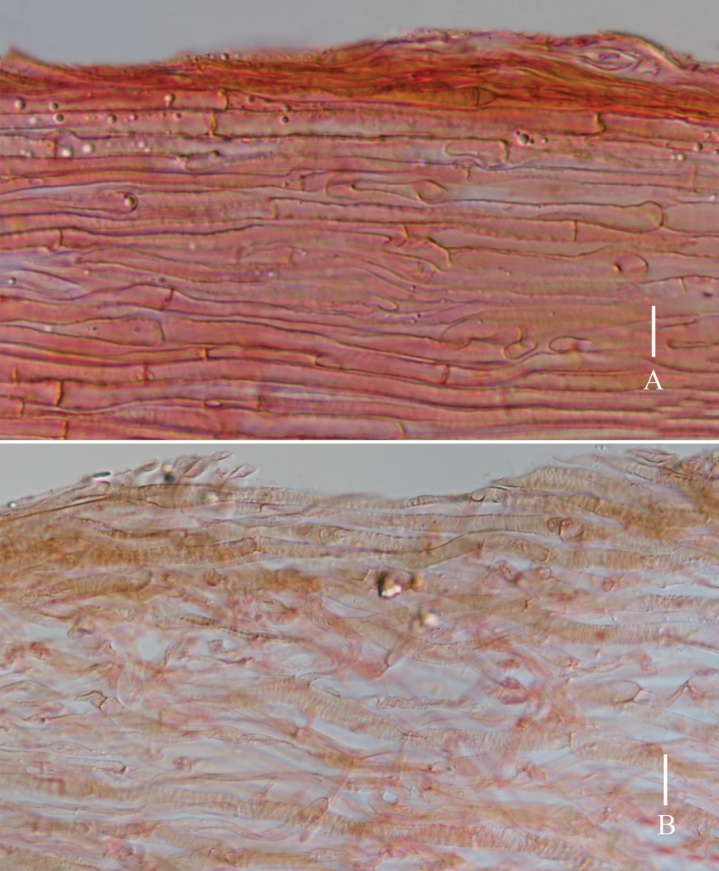
*Lyophyllumshimeji* (L6881) **A** stipitipellis **B** pileipellis. Scale bars: 10 μm.

Figs 7C, D , 10 , 11

##### Description.

***Pileus*** 2.0–3.0 cm diameter, fleshy, fragile, hemispherical, becoming convex with age, abundant black floccus on the surface, dry, dark orange (#756450), slightly depressed of center, deflexed to inflexed of margin; pileus context thick, 0.5–0.7 cm wide, white (#fcfcfc). ***Lamellae*** moderately close together, arcuate, subdecurrent to decurrent, broad, white (#fcfcfc), unchanging color when injured, 3–4 tiers, 0.3–0.4 cm wide, edge even or entire. ***Stipe*** 3.0–5.1 × 1.0–1.4 cm, cylindrical to clavate, grayish yellow (#89877b) on the surface, tapering upwards, smooth; stipe context white (#fcfcfc), unchanging in color when injured. The odor and taste were not distinctive.

***Basidiospores*** [75/2/2] 5.4–7.3 × 4.6–6.6 μm, (Q = 1.0–1.3, Qm = 1.10 ± 0.19), av. 6.03 ± 0.38 × 5.55 ± 0.65 μm, globose, subglobose to broadly ellipsoid, smooth. ***Basidia*** 32–41 × 6–9 μm (N = 20), av. 36.2 ± 3.8 × 8.3 ± 1.15 μm, mostly 4-spored, rarely 2-spored, sterigmata long 3.1–4.5 μm, sometimes with basal clamp connections, clavate, siderophilous granulations. ***Subhymenium*** is composed of moderately thin-walled hyphae, 15–30 μm thick, with 2–3 layers of ovoid, fusiform to narrowly cylindrical hyphae, 5–8 × 3–4 μm. ***Hymenophoral trama*** regular, 120–180 μm wide, consisting of thin and hyaline hyphae, some with clamp connections, narrowly cylindrical hyphal elements 2–4 μm wide. ***Cheilocystidia*** 15–22 (–26) × 3–5 μm, av. 20.6 ± 4.4 × 4.7 ± 1.1 μm, narrowly cylindrical or narrowly clavate, thin-walled. ***Pleurocystidia*** were 16–20 × 3–5 μm in size and av. 18.6 ± 3.7 × 3.7 ± 0.4 μm, narrowly cylindrical or narrowly clavate, thin-walled. ***Pileipellis*** is an interwoven trichodermium composed of almost hyaline interwoven filamentous hyphae, terminal cells 2–4 μm wide, almost cylindrical to subcylindrical, occasional hyphal tips flexuous and sometimes inflated, and some with clamp connections. ***Stipitipellis*** composed of appressed, parallel, thin-walled, 3–6 μm wide. ***Clamp connections*** are present at some septa in the pileipellis, lamellae, and stipitipellis.

##### Habitat.

Clustered in the *Quercus*, *Pinus*, and mixed *Picea* and *Fagus* forests. Known from China, Japan, Sweden, Finland, and Norway (Fujita et al. 1982; Kawai 1997; Yamanaka 2009).

##### Edibility.

This mushroom is highly appreciated and is cultivated worldwide.

##### Additional materials examined.

China • Chongqing Municipality, elev. 1,872 m, 12 October 2023, Tong Lv, HKAS135647; ibid, 12 October 2023, Tong Lv, HKAS135648.

##### Notes.

The originally described *Lyophyllumshimeji* was from Japan as *Tricholomashimeji* Kawam.; it is a famous edible mushroom (Fujita et al. 1982; Kawai 1997; Yamanaka 2009). However, the description of *L.shimeji* is incomplete, lacking both macroscopic and microscopic characteristics. In this study, we meticulously provided the comprehensive and detailed characteristics of *L.shimeji*, enabling more precise and unequivocal identification of this species. This thorough analysis ensures that future taxonomic studies and research endeavors can accurately distinguish *L.shimeji* from other similar fungal species, thereby facilitating a deeper understanding of its ecological role and potential applications in culinary and scientific contexts.

### ﻿*Lyophyllum* cultivation

*Lyophyllum* is a treasure trove of bioactive compounds with significant therapeutic potential (Peterson 2024). All species in this genus are edible and possess medicinal properties, making them valuable in both culinary and pharmaceutical sectors (Zhang et al. 2022a). Two cultivable species, *Lyophyllumshimeji* and *L.decastes*, can be grown using substrates of sawdust and wheat bran, a sustainable and economical method that supports their large-scale cultivation (Thawthong et al. 2014).

The cultivation of *Lyophyllum* mushrooms on sawdust and wheat bran provides a renewable source of these beneficial fungi, supporting the circular economy by utilizing agricultural by-products (corn cob, straw, and wheat bran). This sustainable cultivation method ensures a consistent supply of mushrooms for both consumption and extraction of medicinal compounds, highlighting the versatility and importance of fungi in modern agriculture and healthcare (Pérez-Moreno and Martínez-Reyes 2014).

*Lyophyllum* mushrooms are diverse and edible; *L.shimeji* and *L.decastes* stand out as widely cultivated species. They thrive on sawdust and wheat bran substrates, making them accessible for cultivation (Ohta 1994; Pokhrel et al. 2006). In our study, we used a mixture for cultivating *L.shimeji* and *L.decastes*, which consisted of 80% sawdust, 18% wheat bran, 1% sugar, and 1% plaster.

*Lyophyllumshimeji*, a mushroom species that is both saprophytic and mycorrhizal, is highly valued for its culinary uses, particularly in China and Japan (Imazeki and Hongo 1987; Wang et al. 2020), where it is recognized for its flavor, surpassing that of *Tricholomamatsutake* (S. Ito & S. Imai) Singe. This species is unique in producing fruiting bodies in axenic cultures, facilitating its commercial cultivation (Pérez-Moreno and Martínez-Reyes 2014).

Ohta (1994) revealed that the mycelia of *L.shimeji* grew most rapidly on barley-based synthetic liquid medium. This substrate provides the nutrients necessary for rapid mycelial expansion. Furthermore, fruit-body formation was successfully induced in a medium of barley, beech sawdust, and liquid synthetic nutrients.

**Figure 12.**
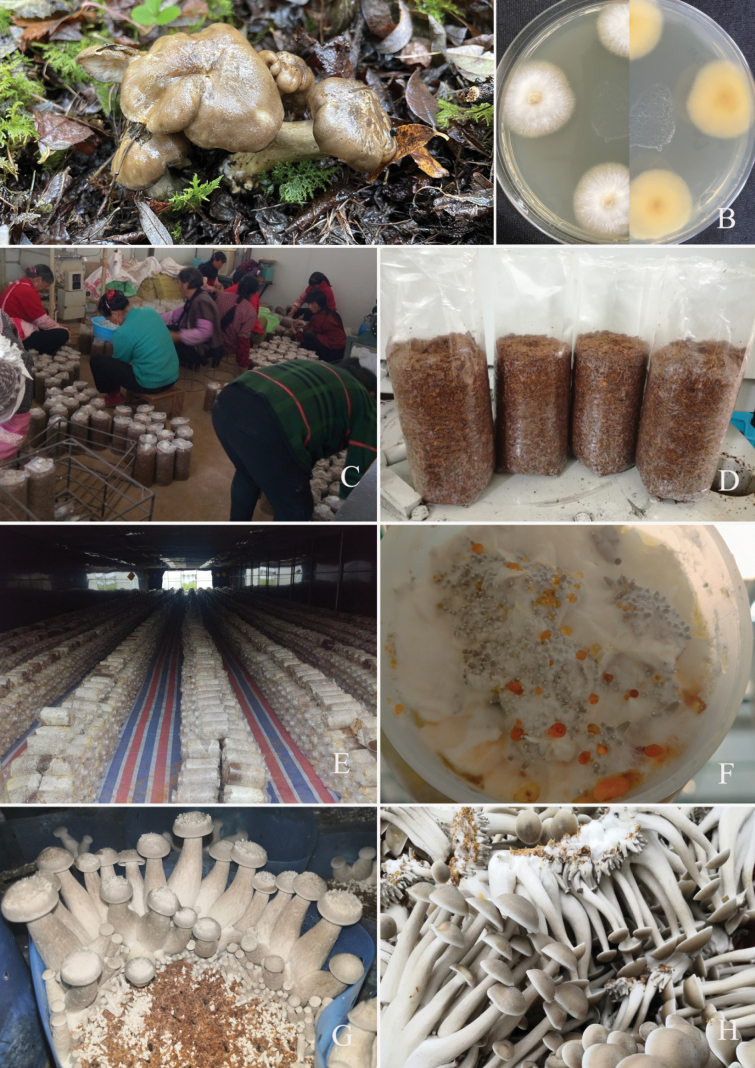
Cultivation process of *Lyophyllumdecastes***A** wild collected *L.decastes***B** culture of *L.decastes***C***L.decastes* bag cultivation **D***L.decastes* grow bags **E** spawn preparation on wood chips **F** primordia of *L.decastes***G***L.decastes* on bag substrate **H** harvest of *L.decastes*.

*Lyophyllumdecastes* is prized for its palatable taste, desirable texture during cooking, and recognized medicinal value. Cultivating *L.decastes* involves a meticulous process (Fig. 12), starting with selecting fermented sawdust from *Quercusaliena* and *Populusdeltoides* as the base substrates (Woo et al. 2009). Dried mushrooms are commonly available in China’s major supermarkets, with a market price range of 80 to 100 RMB/kg. This combination provides essential nutrients to initiate mycelial growth and supports the development of fruiting bodies within a controlled environment, typically within 500 mL bottles. The complexity lies in the fine balance among moisture, temperature, and gas exchange, which must be meticulously managed to prevent contamination and ensure optimal growth.

### ﻿Bioactivities and mode of action of *Lyophyllum*

Polysaccharides extracted from mushrooms are a rich source of bioactive substances. They exhibit a range of biological activities, including anti-tumor and immunomodulatory effects, which have been harnessed in traditional Chinese medicine (Ukawa et al. 2000; Xu et al. 2023). These substances stimulate the immune system, enhancing the body’s defenses against diseases like cancer. The anti-tumor properties are often attributed to specific compounds like β-glucans, which have been shown to activate immune cells and induce an immune response.

*Lyophyllumdecastes*, a species of edible mushroom, has garnered significant attention in the scientific community due to its diverse medicinal properties. Extensive research has underscored its multifaceted therapeutic potential, which includes anti-tumor, anti-hypertensive, anti-diabetic, anti-hyperlipidemic, immunomodulatory, hepatoprotective, and skin lesion protection effects (Kim et al. 1984; Ukawa et al. 2000; Miura et al. 2002; Kokean et al. 2005; Ding et al. 2022; Wang et al. 2022a; Zhang et al. 2022b).

The anti-tumor properties of *L.decastes* have been attributed to its polysaccharide components, particularly β-glucans with β-(1→3) linkages in the main chain and additional β-(1→6) branch points, which are known to enhance the immune response against cancer cells (Wasser 2002; Ren et al. 2012). Xu et al. (2023) have also demonstrated the anti-hypertensive effects of *L.decastes*, with evidence suggesting that its bioactive compounds can help regulate blood pressure.

In the realm of diabetes management, *L.decastes* has shown promise through its ability to modulate glucose metabolism, thereby exhibiting anti-diabetic effects (Kim et al. 1984; Ukawa et al. 2000; Ding et al. 2022). Similarly, its anti-hyperlipidemic properties are linked to the regulation of lipid profiles, which is crucial in preventing cardiovascular diseases. The immunomodulatory effects of *L.decastes* are mediated through its polysaccharides, which can stimulate the immune system, providing a defense against various pathogens. Hepatoprotective effects have been observed in studies where *L.decastes* was found to alleviate liver injury by activating the Nrf2 signaling pathway, thereby reducing inflammation and oxidative stress in the liver. Furthermore, *L.decastes* has been noted for its skin lesion protection effects, which may be beneficial in the treatment of skin conditions and wounds (Xu et al. 2023). These properties are supported by a wealth of scientific studies that have elucidated the underlying mechanisms of action and potential therapeutic applications of *L.decastes* (Dawson et al. 2007).

Polysaccharides found in *L.decastes* have been identified as the primary bioactive compounds responsible for their medicinal benefits (Ding et al. 2022; Wang et al. 2022b; Zhang et al. 2022b). These compounds have been studied for their potential in the treatment of various disease conditions and are considered a significant source of therapeutic agents. Its popularity in China has led to extensive cultivation efforts, making it a significant player in the food and pharmaceutical industries (Zhang et al. 2023).

*Lyophyllum* species exhibit a range of bioactivities and have been studied for their medicinal and nutritional value (Zhang et al. 2023). These mushrooms are known for their immunomodulatory, anti-diabetic, antiviral, antimicrobial, hepatoprotective, and anti-tumor activities (Ukawa et al. 2000; Xu et al. 2023). The bioactive components are primarily polysaccharides and triterpenes, which modulate immune responses and have potential therapeutic applications (Wang et al. 2022a).

## ﻿Discussion

Mushroom production has witnessed a remarkable surge worldwide, with various species cultivated on a large scale. These include *Auricularia* spp., which are known for their jelly-like texture and nutritional value (Ye et al. 2024); *Agaricusbisporus*, commonly referred to as the white button mushroom is favored for its meaty texture (Young et al. 2024); and *Grifolafrondosa* is esteemed for its medicinal properties and umami flavor (Sun et al. 2023; Tang et al. 2024). Culturing these mushrooms has not only met the demands of a health-conscious consumer base but has also contributed significantly to the global food industry (Li and Xu 2022). The versatility of these mushrooms for various culinary applications, from everyday meals to gourmet cuisine, has fueled their widespread production. Moreover, the environmental benefits of mushroom cultivation, such as converting agricultural waste into a valuable food source, have further propelled the industry’s growth and inspired a shift towards more sustainable practices in the food industry (Bakratsas et al. 2021).

The genome of *L.shimeji* has been sequenced, revealing insights into its evolutionary history and providing a foundation for future research to enhance its cultivation and culinary qualities (Kobayashi et al. 2023). Its status as a facultative fungus, capable of existing both as a decomposer and in symbiosis with plant roots, makes it ecologically versatile. Cultivating *L.shimeji* is a sustainable practice that enhances soil health, contributes to nutritional security, and promotes environmental sustainability.

*Lyophyllumdecastes*, known as “Luronggu” in China, is a culinary and medicinal mushroom with a rich flavor and desirable texture (Zhang et al. 2023). It is highly valued for its nutritional content and is widely cultivated in China, particularly in Shandong, Jiangxi, Shanghai, and Hebei Provinces (Zhang et al. 2023). This mushroom not only offers a delightful taste experience but also boasts a range of pharmacological activities, such as antioxidation, hypolipidemic, antidiabetic, and antiproliferative properties (Wang et al. 2022a). The fruiting body of *L.decastes* is traditionally used for its medicinal compounds, including polysaccharides, which exhibit significant therapeutic potential (Zhang et al. 2023).

In morphology, species of *Lyophyllum* exhibit variability; the basidiospores include both globose and broadly fusiform shapes. Some species of basidiomata turn black when injured (Lyu et al. 2024), while others remain unchanged. The genus Lyophyllum has been divided into two subgenera (subgen. Lyophyllum and subgen. Lyophyllopsis) and three sections (sect. Carneoviolacei, sect. Lyophyllum, and sect. Semitalini). However, these results were not supported by phylogenetic analysis and need to be verified by collecting more specimens in the future.

In this study, we combined sequences of four non-translated loci (5.8 S, LSU + ITS1 + ITS2, *tef*1-α exon + *rpb*2 exon, and *tef*1-α intron+*rpb*2 intron) to carry out phylogenetic analyses of *Lyophyllum* species. We investigated the phylogenetic relationships between the two novel edible mushrooms and two known edible mushrooms. Twenty *Lyophyllum* specimens have been studied, with ten specimens from a previous study and ten new collections providing additional genetic data.

Over the last decade, research on *Lyophyllum* species diversity has often relied on phylogenetic analyses based solely on the internal transcribed spacer (ITS) region or a combination of ITS and LSU of the ribosomal RNA gene (Li et al. 2023, 2024; Wei et al. 2023). However, these approaches are insufficient to accurately depict the phylogenetic relationships among different clades within *Lyophyllum*. Our study employed a multi-gene analysis incorporating the ITS, LSU, *rpb*2, and *tef*1-α genes to address this limitation. This comprehensive approach has allowed for a more precise representation of the phylogenetic relationships between *Lyophyllum* species, thus enhancing our understanding of their evolutionary history and diversification.

## Supplementary Material

XML Treatment for Lyophyllum edulis

XML Treatment for Lyophyllum pallidofumosum

XML Treatment for Lyophyllum sinense

XML Treatment for Lyophyllum shimeji

## ﻿Additional information

### Author ORCIDs

Song-Ming Tang https://orcid.org/0000-0002-6174-7314

Feng-Ming Yu https://orcid.org/0000-0001-9133-8645

Samantha C. Karunarathna https://orcid.org/0000-0001-7080-0781

Zong-Long Luo https://orcid.org/0000-0001-7307-4885

Lin Li https://orcid.org/0009-0000-8167-2965

### Data availability

All of the data that support the findings of this study are available in the main text.
